# Supplementary material for: Variants Tagging LGALS-3 Haplotype Block in Association with First Myocardial Infarction and Plasma Galectin-3 Six Months after the Acute Event
Source: Genes (Basel). 2022 Dec 29;14(1):109. doi: 10.3390/genes14010109 (PMC9859409; doi:10.3390/genes14010109)
Supplement: Supplementary file 1 [file genes-14-00109-s001.zip › genes-2028861-supplementary.pdf]

Supplementary files

Table S1. Genotype and allele frequencies of variants rs4040064, rs11628437 and rs7159490 in controls and patients with myocardial infarction.

Variant	Controls, N (%) N=323	Patients, N (%) N=546	*p* value
**rs4040064**			
genotypes			
GG	164 (50.77)	288 (52.75)	
GT	131 (40.56)	222 (40.66)	0.57
TT	28 (8.67)	36 (6.59)	
allele frequencies			
G	0.71	0.73	0.36
T	0.29	0.27	
**rs11628437**			
genotypes			
GG	126 (39.01)	208 (38.10)	
GA	164 (50.77)	265 (48.53)	0.41
AA	33 (10.22)	73 (13.37)	
allele frequencies			
G	0.64	0.62	0.40
A	0.36	0.38	
**rs7159490**			
genotypes			
CC	266 (82.35)	444 (81.32)	0.86
CT+TT	57 (17.65)	102 (18.68)	
allele frequencies			
C	0.91	0.90	0.62
T	0.09	0.10	

Pearson's Chi-square (χ^2) test was used for the comparison of the allele frequencies and genotype distribution between controls and patients with first acute myocardial infarction and p values <0.017 were considered statistically significant.

Rs7159490 genotypes are presented according to the model CC vs. CT+TT, since TT genotype was present in only 1 control subject and 4 MI patients, and Chi-square test is inaccurate when expected numbers are less than 5.

Table S2. General characteristics of patients with the first myocardial infarction included in the expression analysis.

Variable	MI group, N=92
Age, years	55.2 ± 8.0
Gender, f/m, %	18.5/81.5
BMI, kg/m2	27.24 ± 3.84
TC, mmol/l	5.50 ± 1.05
HDLC, mmol/l	1.09 ± 0.27
LDLC, mmol/l	3.55 ± 0.99
TG, mmol/l	1.85 ± 1.17
T2DM, %	34.8
Hypertension, %	51.1
Current smokers, %	60.1
Aspirin, %	100
Clopidogrel, %	98.9
LMWH, %	96.7
UFH, %	64.1
Nitrates, %	94.6
ACE inhibitors, %	97.8
Beta-blockers, %	78.3
Diuretics, %	18.5
Statins, %	97.8

Values are mean ± SD for: Body Mass Index (BMI), age, Total Cholesterol (TC), Triglycerides (TG), High-Density Lipoprotein Cholesterol (HDLC) and Low-Density Lipoprotein Cholesterol (LDLC). T2DM – type 2 diabetes mellitus. LMWH – Low Molecular Weight Protein; UFH – UnFractionated Heparin.

Table S3. General characteristics of patients with the first myocardial infarction included in the quantification of plasma Gal-3 levels.

Variable	MI group, N=189
Age, years	55.5 ± 8.3
Gender, f/m, %	24.9/75.1
BMI, kg/m2	27.5 ± 3.9
TC, mmol/l	5.58 ± 1.14
HDLC, mmol/l	1.08 ± 0.26
LDLC, mmol/l	3.66 ± 1.05
TG, mmol/l	1.89 ± 1.13
T2DM, %	35.9
Hypertension, %	58.2
Current smokers, %	62.4
Aspirin, %	99.3
Clopidogrel, %	99.3
LMWH, %	95.8
UFH, %	63.4
Nitrates, %	95.8
ACE inhibitors, %	95.1
Beta-blockers, %	82.4
Diuretics, %	16.2
Statins, %	98.6

Values are mean ± SD for: Body Mass Index (BMI), age, Total Cholesterol (TC), Triglycerides (TG), High-Density Lipoprotein Cholesterol (HDLC) and Low-Density Lipoprotein Cholesterol (LDLC). T2DM – type 2 diabetes mellitus. LMWH – Low Molecular Weight Protein; UFH – UnFractionated Heparin.

Table S4. Main characteristics of MI patients categorized according to the cut-off plasma Gal-3 value*.

Variable	pGal-3 ≤17.8 ng/mL	pGal-3 >17.8 ng/mL	*p* value
**Overall**	N=107	N=82	
Age, year	55.5 ± 7.6	54.7 ± 8.6	ns§
Gender, female, %	12.8	48.5	<0.0001
NYHA class III and IV, %	2.7	12.1	0.03
Systolic dysfunction, %	20.0	29.0	ns
Δ LVEDV, mL	1.97 ± 21.41	3.10 ± 25.58	ns§
Δ LVESV, mL	0.95 ± 16.58	2.51 ± 19.02	ns§
Δ LAD, mm	0.51 ± 4.46	2.07 ± 3.88	0.04§
BMI, kg/m^2	26.74 ± 2.97	28.76 ± 4.69	0.005§
**Males**	N=98	N=44	
Age, year	54.9 ± 7.7	52.3 ± 8.4	ns§
NYHA class III and IV, %	3.1	4.5	ns
Systolic dysfunction, %	18.4	31.8	ns
Δ LVEDV, mL	0.27 ± 21.56	5.75 ± 30.09	ns§
Δ LVESV, mL	0.13 ± 16.82	5.45 ± 22.40	ns§
Δ LAD, mm	0.56 ± 4.03	2.10 ± 3.68	ns§
BMI, kg/m^2	26.91 ± 2.84	30.27 ± 4.45	0.0002§
**Females**	N=12	N=35	
Age, year	55.5 ± 7.6	54.7 ± 8.6	ns§
NYHA class III and IV, %	0	20.0	ns
Systolic dysfunction, %	33.3	25.7	ns
Δ LVEDV, mL	10.72 ± 19.78	0.24 ± 18.60	ns§
Δ LVESV, mL	5.26 ± 16.31	-1.56 ± 13.24	ns§
Δ LAD, mm	-0.04 ± 7.62	2.17 ± 4.15	ns§
BMI, kg/m^2	24.30 ± 2.64	27.81 ± 4.63	0.02§

* – Based on the US Food and Drug Administration-cleared cut-off point for HF risk stratification (≤17.8 ng/mL low risk, 17.9-25.9 ng/mL intermediate risk and >25.9 ng/mL high risk), we set the threshold for Gal-3 plasma at 17.8 ng/mL.

Values are mean ± SD for age, body mass index (BMI), change of left ventricular end-diastolic volume (Δ LVEDV), left ventricular end-systolic volume (Δ LVESV) and left atrial dimension (Δ LAD).

NYHA – *New York Heart Association* classification.

Systolic dysfunction was defined as LVEF <40%.

§– Mann-Whitney *U* test was used to compare the values of continuous variables with a skewed distribution between patients with plasma Gal-3 below and above 17.8 ng/mL.

Pearson's Chi-square (χ^2) test was used for comparison of the categorical variables.

p values <0.05 were considered statistically significant; ns – non significant.

Table S5. Plasma Gal-3 levels in patients six months post-MI according to the haplotypes inferred from the variants rs4040064, rs11628437 and rs7159490, in females.

Haplotype[§]	Haplotype frequency	Mean pGal-3 [95% CI], ng/mL	*p* value
Females, N=47			
GGC	0.771429	39.8 [26.3-53.4]	referent haplotype
TAC	0.171429	52.8 [27.2-78.3]	0.43
GAT	0.042857	47.3 [5.9-88.8]	0.76
GAC	0.014286	34.2 [-63.6-132.1]	0.91

[§] – The alleles in haplotypes are in the following order: rs4040064 G/T, rs11628437 G/A and rs7159490 C/T.

p values were corrected for multiple testing and values <0.017 were considered statisticaly significant.

Table S6. Association of rs4040064, rs11628437 and rs7159490 haplotypes with a change of echocardiographic parameters within six months post-MI in males.

Cardiac parameter	Haplotype frequency	Means [95% CI]	*p* value
Δ LV End-diastolic volume (mL)			
GGC	0.52079	5.52 [-1.69 – 12.73]	ref. haplotype
TAC	0.328629	-3.73 [-16.17 – 8.71]	0.27
GAT	0.079397	2.81 [-22.15 – 27.78]	0.84
GAC	0.042866	-6.85 [-40.67 – 26.97]	0.48
Δ LV End-systolic volume (mL)			
GGC	0.520795	3.25 [-1.68 – 8.19]	ref. haplotype
TAC	0.328621	-3.03 [-12.30 – 6.23]	0.29
GAT	0.07921	16.63 [2.19 – 31.08]	0.09
GAC	0.043062	-9.11 [-43.76 – 25.53]	0.49
Δ LV Ejection fraction (%)			
GGC	0.524566	1.37 [-0.73 – 3.46]	ref. haplotype
TAC	0.322846	3.02 [-0.38 – 6.43]	0.48
GAT	0.082719	-7.81 [-12.61 – -3.01]	0.001
GAC	0.041803	8.95 [-1.14 – 19.04]	0.16
Δ Left atrial dimension (mm)			
GGC	0.524351	2.02 [0.97 – 3.07]	ref. haplotype
TAC	0.322741	0.05 [-1.90 – 2.01]	0.12
GAT	0.082399	-1.24 [-5.87 – 3.38]	0.18
GAC	0.042228	-1.15 [-13.47 – 11.16]	0.62
Δ Global radial strain (%)			
GGC	0.533337	4.44 [1.62 – 7.26]	ref. haplotype
TAC	0.31886	0.73 [-4.42 – 5.89]	0.29
GAT	0.077919	-3.85 [-10.45 – 2.76]	0.03
GAC	0.050895	5.66 [-7.42 – 18.74]	0.86
Δ Stroke volume (ml)			
GGC	0.521995	9.52 [3.56 – 15.48]	ref. haplotype
TAC	0.326743	0.24 [-10.19 – 10.67]	0.18
GAT	0.079805	-16.76 [-41.56 – 8.04]	0.04
GAC	0.041071	-4.93 [-33.55 – 23.70]	0.33

Values are shown as expected phenotypic mean with its 95% confidence interval (CI). *P* values were corrected for multiple testing and values ≤0.005 were considered statistically significant. Δ – Change from 3-5 days to six months.

Figure S1. Relative *LGALS-3* mRNA expression in PBMCs from patients 6 months post-MI in regard to the haplotypes of the variants rs4040064, rs11628437, and rs7159490, in females.

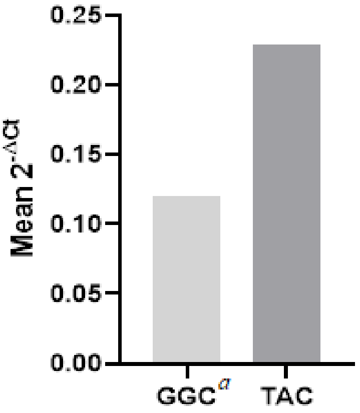

Haplotype	Haplotype frequency	Mean $2^{-\Delta Ct}$ [95% CI]	*p* value
GGC[a]	0.764706	0.120 [0.024-0.216]	
TAC	0.205882	0.229 [0.130-0.329]	0.23

Quantitative real-time PCR was performed on 17 cDNA of human PBMCs to quantify the relative gene expression of *LGALS-3* and *Cyclophilin A*. Relative *LGALS-3* mRNA expression in regard to rs4040064, rs11628437, and rs7159490 variant haplotypes was assessed using THESIAS v3.1 software. Results are reported as the mean $2^{-\Delta Ct}$ value with its 95% confidence interval (CI) for each haplotype, where the ΔCt value is the difference between the Ct value of *LGALS-3* and the Ct value of *Cyclophilin A* for each sample. No significant association was found between the haplotypes inferred from rs4040064, rs11628437, and rs7159490 and relative *LGALS-3* mRNA expression in MI patients 6 months post-MI. The haplotypes GAC, TGC, GGT, TAT, and TGT with an estimate frequency <0.01 were not included in the statistical analyses. The haplotype GAT was not presented since the standard error of the mean value of $2^{-\Delta Ct}$ was high, making the result meaningless. [a] – referent haplotype set by the Thesias software. *P* values were corrected for multiple testing, and values <0.017 were considered statistically significant.
